# A networks method for ranking microRNA dysregulation in cancer

**DOI:** 10.1186/1752-0509-7-S5-S3

**Published:** 2013-12-09

**Authors:** William T Budd, Sarah Seashols, Danielle Weaver, Cyriac Joseph, Zendra E Zehner

**Affiliations:** 1 Virginia Commonwealth University Department of Biochemistry and Molecular Biology, 1101 East Marshall Street, Richmond, VA 23298-0037, USA; 2 Virginia Commonwealth University Department of Forensic Science, Richmond, VA 23298-0037, USA; 3 Virginia Commonwealth University Center for the Study of Biological Complexity, Richmond, VA 23284-3079, USA; 4 Massey Cancer Center, 401 College Street, Richmond, VA 23298, USA

**Keywords:** miRNA, prostate cancer, networks biology, systems biology

## Abstract

**Background:**

Despite the lack of agreement on their exact roles, it is known that miRNAs contribute to cancer progression. Many studies utilize methods to detect differential regulation of miRNA expression. It is prohibitively expensive to examine all potentially dysregulated miRNAs and traditionally, researchers have focused their efforts on the most extremely dysregulated miRNAs. These methods may overlook the contribution of less differentially expressed but more functionally relevant miRNAs. The purpose of this study was to outline a method that not only utilizes differential expression but ranks miRNAs based on the functional relevance of their targets. This work uses a networks based approach to determine the sum node degree for all experimentally verified miRNA targets to identify potential regulators of prostate cancer initiation, progression and metastasis.

**Results:**

Here, we present a method for identifying functionally relevant miRNAs that contribute to prostate cancer development. This paper shows that miRNAs preferentially regulate highly connected, central proteins within a protein-protein interaction network. Known targets of miRNAs differentially regulated during prostate cancer progression are enriched in pathways with known involvement in tumorigenesis. To demonstrate the applicability of our method, we utilized a unique model of prostate cancer progression to identify five miRNAs that may contribute to the oncogenic state of the cell. Three of these miRNAs have been shown by other studies to have a role in cancer but their exact role in prostate cancer remains undefined.

**Conclusion:**

Developing methods to determine which miRNAs to carry forward into biological and biochemical analyses is important as traditional approaches often overlook miRNAs that contribute to oncogenesis. Our method applied to a model of prostate cancer progression was able to identify miRNAs with roles in prostate cancer development.

## Background

Prostate cancer is a major medical problem for men around the world. According to the American Cancer Society, it is the most common non-cutaneous malignancy in men [1]. Nearly 250,000 men will be diagnosed with prostate cancer this year and it is estimated that 35,000 will ultimately succumb to the disease. Successful treatment depends upon early identification, as death rates increase significantly and treatment options decline when the tumor leaves the confines of the prostate gland [2]. The most significant event during prostate cancer progression is metastatic dissemination [3]. Despite this significance, molecular events surrounding tumor progression and metastasis are poorly understood.

In recent years, our knowledge of microRNAs (miRNA) has evolved and it is now apparent that miRNAs are an important class of non-coding RNA that regulates the proteome [4]. miRNAs are short nucleotide sequences (20-22 nucleotides in length) that alter gene expression by binding to target mRNAs and either repressing translation or promoting mRNA cleavage. In the presence of external cues and environmental stressors, miRNAs can induce rapid changes in the proteome, allowing the cell to respond in a more precise and energy efficient manner [5]. Numerous cellular processes are affected by miRNA, including differentiation, growth/hypertrophy, cell cycle control and apoptosis [6]. Aberrant expression of miRNAs has been shown to contribute to the development of many pathological conditions including cancers of the breast, prostate, thyroid, and B-cell lymphomas [7]. Considering their ability to modify gene expression through direct action on mRNA the analysis of miRNAs is an important emerging field of study in decoding the genome, its epigenetic modification, and its regulation. Though miRNAs have been casually observed to be associated with prostate cancer, there is no clear consensus as to which specific miRNAs contribute to oncogenesis and ultimately metastasis.

Many miRNA genes are dysregulated in cancer and influence tumor formation/ progression because they are located in fragile regions of the genome that are commonly overexpressed, deleted or epigenetically modified [8]. Dysregulated miRNAs have been shown to contribute to oncogenesis by the loss of tumor suppressing miRNAs or increased expression of oncomiRs [9]. Both loss of tumor suppressors and increase of oncomiRs can ultimately result in increased cell growth, proliferation, invasiveness or metastasis. Aberrant expression of even a single miRNA has the potential to influence a large number of cellular processes, since it is predicted that each miRNA has the potential to affect hundreds of proteins. Thus, miRNA dysregulation can destabilize homeostatic balance by affecting levels of a multitude of target proteins.

Although it is clear that disturbance of miRNA expression can influence tumorigenesis, there is little agreement on specific miRNAs that contribute to the pathogenesis and metastasis of the prostate tumor. Many studies have attempted to characterize a signature that can identify malignant prostate tissue from its benign counterpart but generally have failed to reveal a consistent signature capable of discriminating between phenotypes. Traditional methods typically examine the most significantly, differentially expressed miRNA. However, such analyses can overlook the emerging potential that miRNAs can exert on downstream proteins. It is reasonable to suspect that smaller expression changes can have a greater influence toward tumorigenicity, if they regulate important protein targets. As each miRNA can influence the level of numerous protein targets, even slightly dysregulated miRNAs can exert a large effect on cellular behavior.

Cancer is the end result of numerous alterations in biochemical pathways and networks [10]. Understanding the molecular perturbations that underlie cancer initiation, progression and metastasis are critical. Systems biologists seek to gather information about multiple types of molecules (genes, proteins, RNAs) in the cell and integrate the information in order to understand the perturbations underlying a given pathology from a broader perspective. Complex interactions can be modeled as a biological network with the macromolecule represented as a node and interactions modeled as edges [5]. Network properties are described mathematically and their contributions to homeostasis are estimated. An important indicator of molecular importance is node degree. A highly connected node is more likely to be essential and cause disease when dysregulated [11,12]. The local connectivity and position of a protein within the global network can be used to identify key proteins that are likely to cause disease when aberrantly expressed. The purpose of this paper is not to elucidate specific miRNAs that drive prostate cancer development but rather outline a method for ranking differentially expressed miRNAs. This work shows that expression profiling of a prostate cancer progression model in association with networks biology has the potential to reveal more relevant miRNAs that drive prostate tumor progression.

## Results and discussion

### miRNAs regulate highly connected protein nodes and target pathways involved in cancer

**Figure 1:**
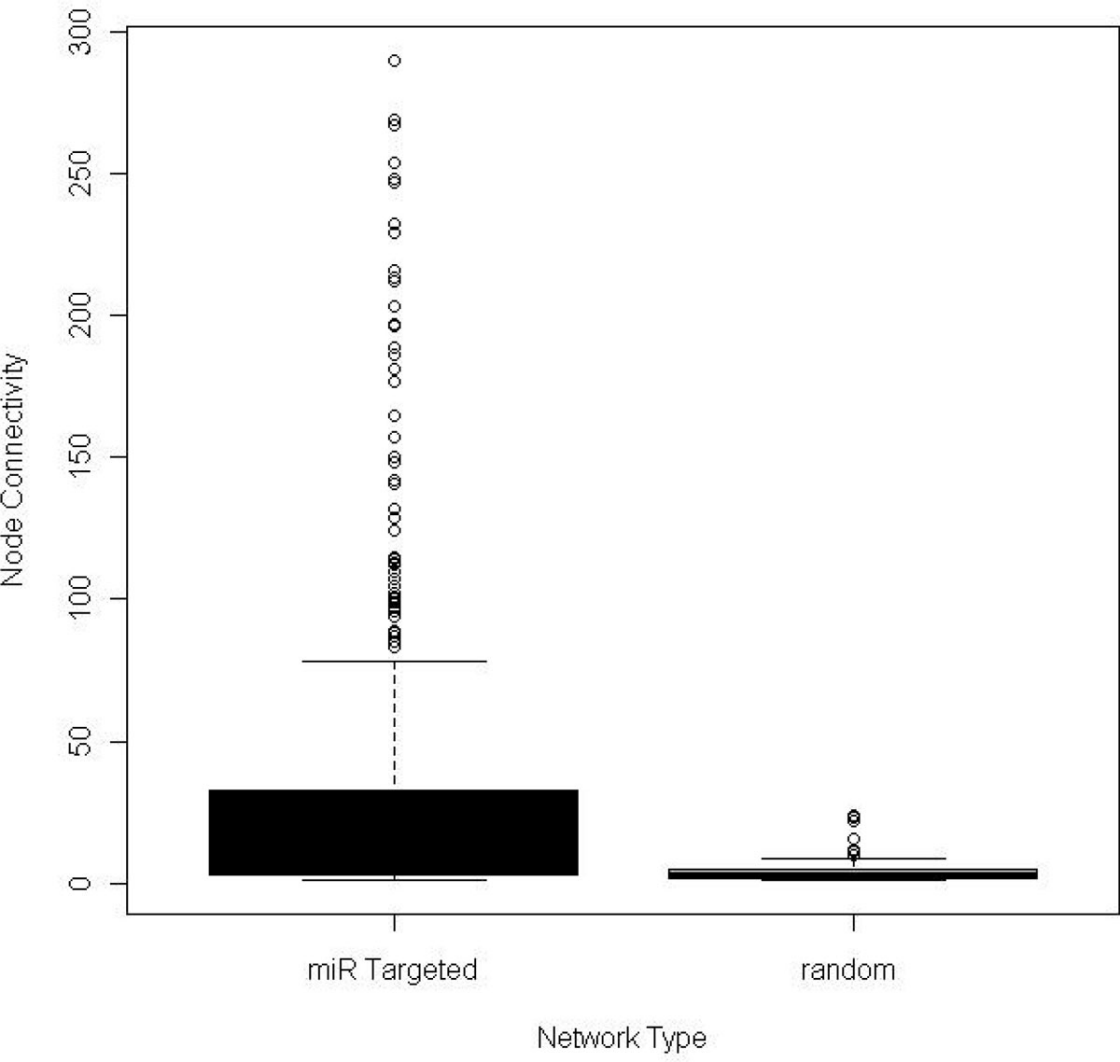
**Connectivity of miRNA targets**. Two shortest path protein-protein interaction networks were built using the Agilent literature search function within Cytoscape 2.8 and topological measures evaluated using CenstiScaPe 2.76. The first network was built using proven targets of miRNAs that are dysregulated during the development of prostate cancer. The other network was built from randomly chosen proteins that are expressed in the prostate but chosen without regard to miRNA status. A whisker plot composed using R displays the differences in the mean node degree between the two PPI networks.

**Figure 2:**
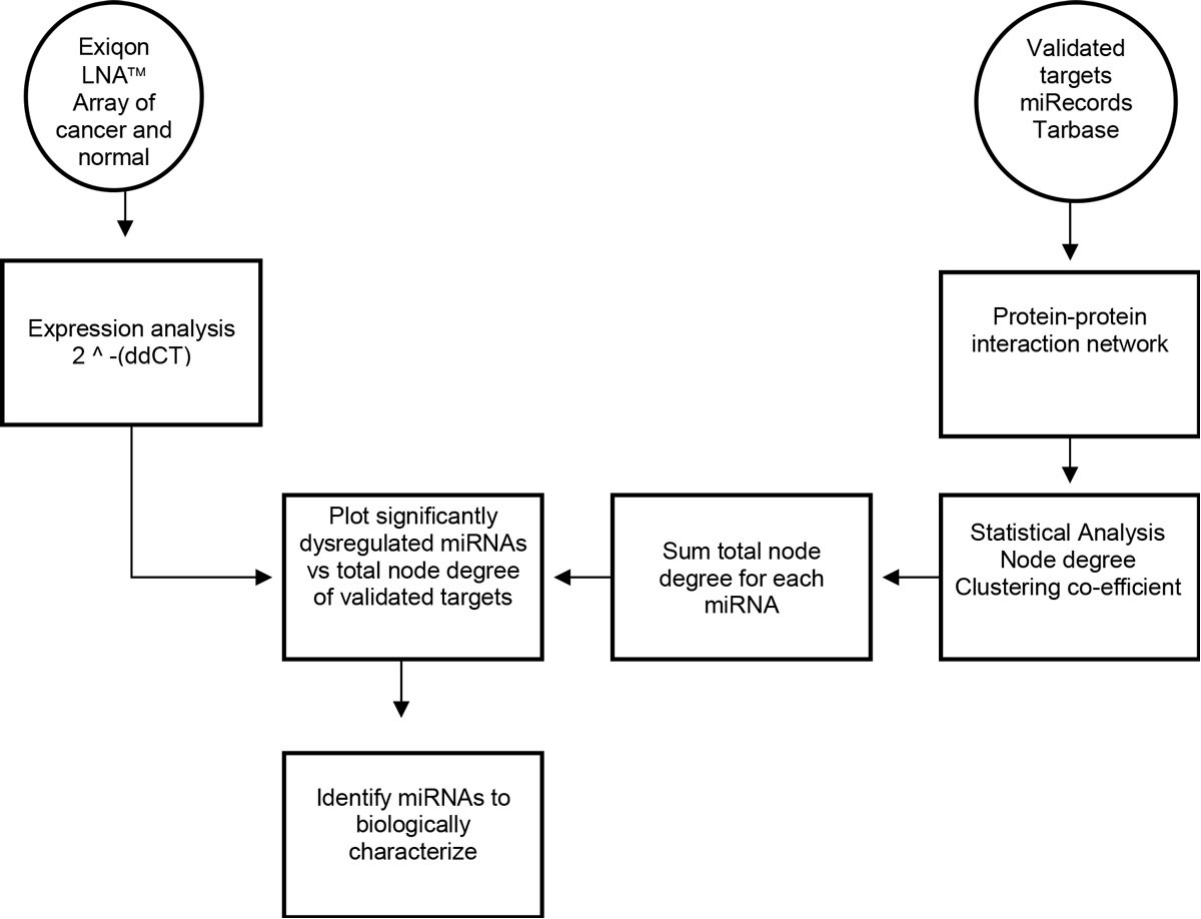
**Outline of project design**. The method outlined in this paper integrates information obtained about validated targets of miRNAs dysregulated in prostate cancer in order to rank differentially expressed miRNAs. Node degrees for each miRNA are determined and used to rank which miRNAs to carry forward to biological characterization.

A protein-protein interaction network (PPI) was built from proven targets of known dysregulated miRNAs involved in carcinogenesis of the prostate. This network was used to determine whether or not miRNAs have a tendency to regulate highly connected protein nodes. Comparing the average node of proteins within a PPI network built from proteins chosen at random to a network of proteins regulated by miRNAs reveals significant differences in the average node degree, closeness centrality and network stress (p-value <0.0001) (Table 1). This analysis shows that miRNAs preferentially target highly connected protein nodes that are generally considered to be key factors within the cell [13]. Studies have shown that proteins with a high node degree and lower closeness centrality are more likely to cause lethality when dysregulated [14]. The average protein in the miRNA targeted network has a mean node degree of nearly 30, while the randomly chosen protein network has a mean node degree of less than five (Figure 1). The observation that miRNAs tend to preferentially regulate highly connected proteins can be used to estimate the contribution of a miRNA to the overall stability within a cell. Our method outlined in Figure 2, begins by building a data table of each miRNA and associating it with proven mRNA targets. Each miRNA/mRNA interaction is paired with the PPI degree of the mRNA target. This approach will be used to functionally estimate the contribution of miRNA dysregulation to tumorigenic potential.

**Table 1:** 

Descriptor	Prostate Cancer miRNA Targets	Randomly Chosen Prostate Proteins
Mean degree	29.80	4.46
Closeness	.000496	.1258
Stress	28,251.6	5821

**Figure 3:**
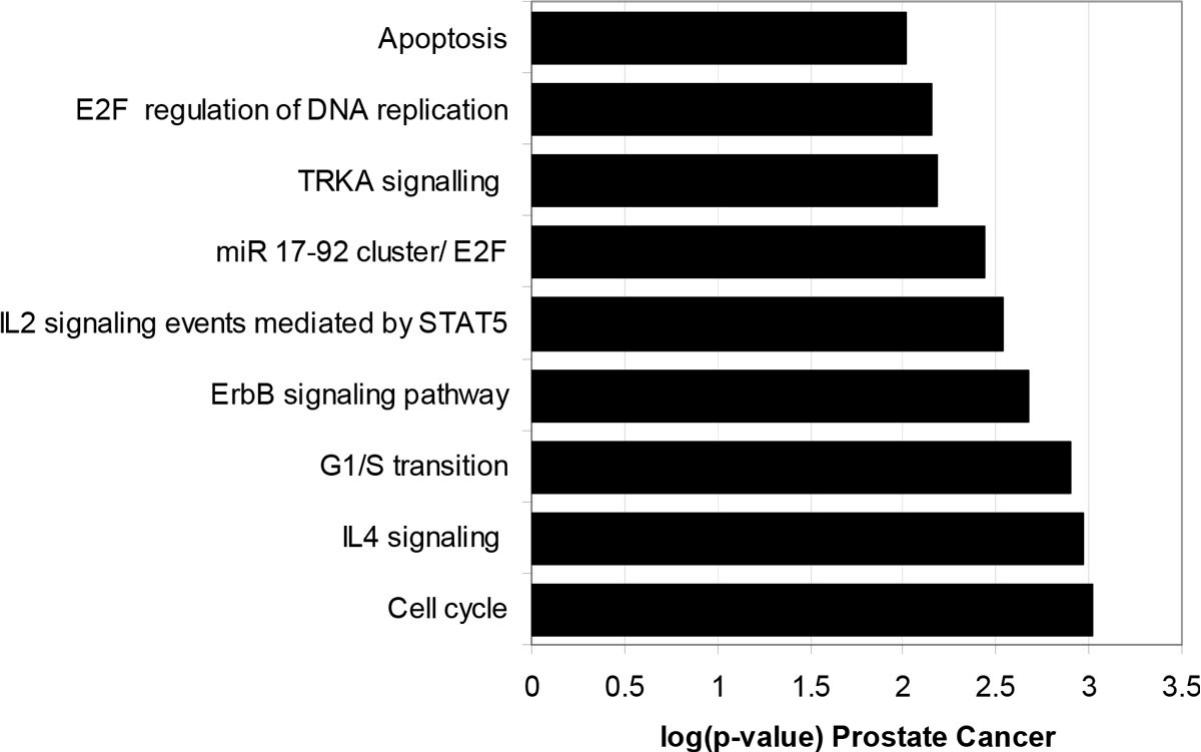
**ToppCluster pathway analysis of known prostate cancer miRNA targets**. Validated targets of dysregulated miRNAs were clustered into pathways using ToppCluster. Corrections for multiple comparisons was accomplished using a Bonferroni correction and statistical significance was set at p-value < 0.05. The log base 10 of the p-value is displayed. Log transformed p-values greater than 1.5 are statistically significant.

ToppCluster pathway analysis of known targets of miRNAs dysregulated during oncogenesis reveals an enrichment of proteins involved in pathways associated with cancer (Figure 3) [15]. A similar analysis did not identify any pathway enrichment in the list of randomly chosen proteins. It is well known that driving cell cycle progression or inhibiting apoptosis can promote neoplastic transformation [16]. Many of the miRNA targets are key regulators of pathways that lead to uncontrolled cell proliferation and survival. The pathways along with key protein members are described in Table 2.

**Table 2:** 

Pathway Name	Description	Relevant miRNA Targeted Proteins
Apoptosis	Process of programmed cell death	APAF1, BAK1, BCL2, BNIP3L, CASP6, CASP7, CDKN2A, FADD, FAS, IGF1R, JUN, MCL1, MYC, PIK3R1

E2F regulation of DNA replication	E2F family of transcription factors affects cell cycle progression, apoptosis and DNA synthesis.	CCNE1, CDC25A, DHFR, E2F1, E2F2, E2F3, PRIM1, RB1, TYMS

TRKA signaling	Activation leads to cell survival and replication	ADCY6, AKT2, CDKN1A, CDKN1B, CRK, FOXO1, IRS1, KRAS, MAPK12, MAPK14, MAPK7, MTOR, NRAS, PIK3R1, PTEN, RHOA

miR-17-92 cluster/ E2F	Regulation of E2F and Myc by members of miR-17-92 cluster	E2F1, E2F2, E2F3, MYC

IL2 signaling events mediated by STAT5	Cytokine signaling pathway involved in immune response to foreign infection	BCL2, CCNA2, CCND3, CDK6, FOXP3, JAK1, MYC, PIK3R1, SP1

ErbB Signaling	ErbB family of receptor tyrosine kinases regulates motility, survival, apotosis, proliferation	CCND1, CDKN1A, CDKN1B, CRK, EGFR, ERBB2, ERBB3, FOXO1, JUN, KRAS, MTOR, MYC

G1/S Transition	Cell cycle checkpoint	CCND1, CDK4, CDKN1A, CDKN1B, CDKN2A, E2F1, E2F2

IL-4 Signaling	Regulates immune response signaling including B cell proliferation, T and B cell survival, production of immunoglobulins, and chemokine production.	BCL2, CCNA2, CCND3, CDK6, FOXP3, JAK1, MYC, PIK3R1, SP1

Cell Cycle	Cell division, replication, and maturation.	CCNA2, CCND3, CCNE1, CDC14B, CDC25A, CDK4, CDK6, CDKN1A, CDKN1B, CDKN2A, E2F1, E2F2, E2F3, PLK1, RB1

### Expression profiling of prostate cancer progression model

Many model cell lines have been used to explore prostate cancer progression. Most are derived from metastatic sites and thus may not represent the best model for elucidation of early indicators of cancer formation [17-19]. The cells utilized in this study were obtained from normal prostate tissue immortalized with SV40 large T antigen (P69) and cycled through male athymic nude mice to obtain the highly tumorigenic and metastatic variant (M12) [20]. This unique, isogenic model may provide insights into the molecular causes that initiate cancer formation that may be missed in other prostate cancer cell lines established from end stage, metastasized tumors.

**Figure 4:**
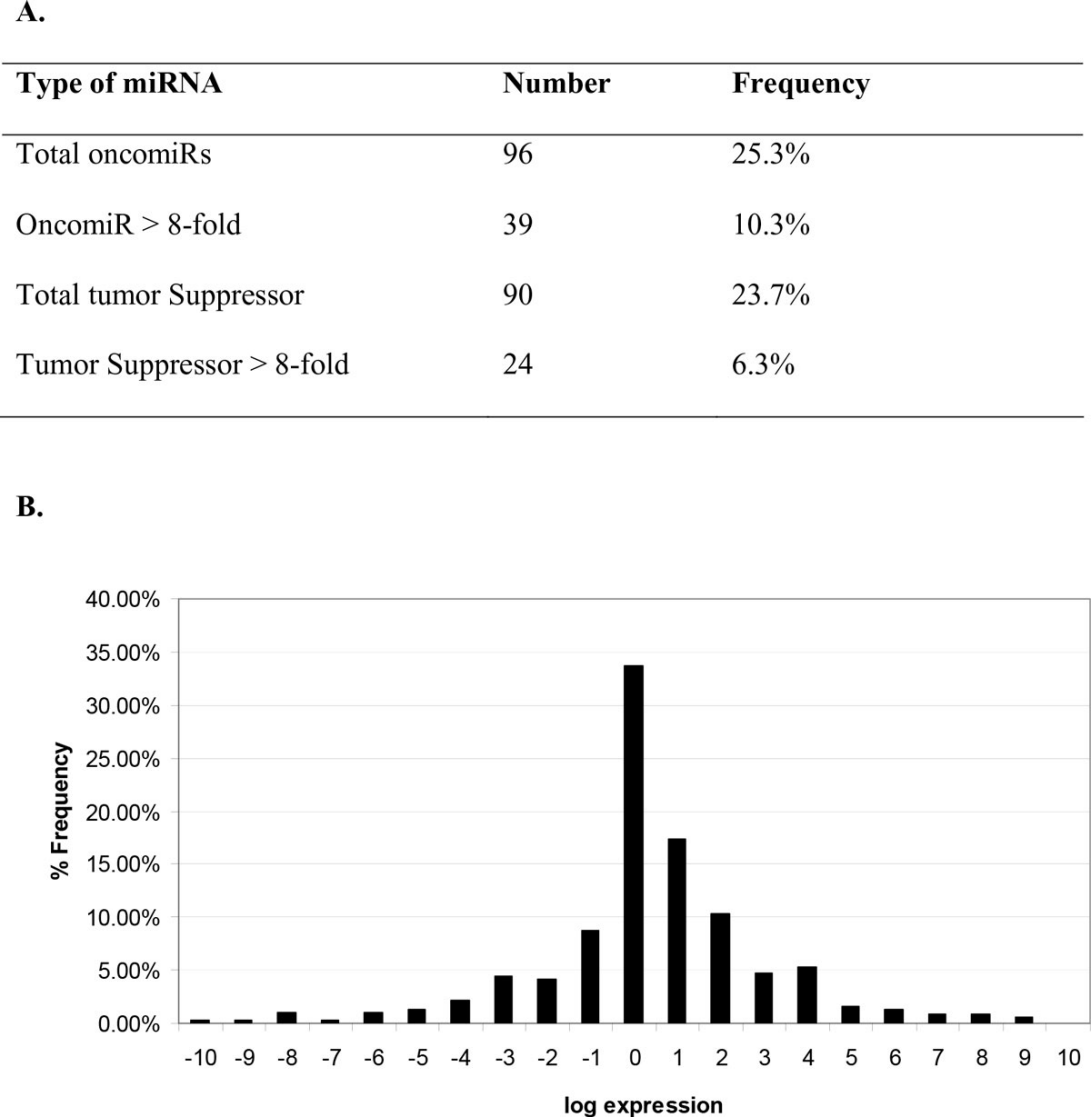
**A: Frequency distribution of qRT-PCR array of prostate cancer cell lines**. Two variants (P69 and M12) of a genetically related prostate cancer progression model were compared using the Exiqon miRCURY LNA™ Universal RT microRNA PCR system (Exiqon, Denmark). miRNA expression was calculated by the 2^-ddCT method [43]. The total number and frequency of potential oncomiRs and tumor suppressors is displayed as well as the subset with an expression difference of greater than 8-fold (A). **Figure 4-B: Frequency distribution of qRT-PCR array of prostate cancer cell lines**. miRNAs functioning as oncomiRs demonstrated higher expression levels in the M12 variant and have a log transformed ratio greater than 1.0. Tumor suppressing miRNAs have a log transformed expression ratio of less than 1.0. A relative frequency distribution of log transformed miRNA fold changes was created using Microsoft Excel (B).

We discovered 186 miRNAs that significantly change (>2-fold) from the parental P69 to the highly tumorigenic M12 cell line (Figure 4A). Ninety miRNAs were lost as the tissue became more tumorigenic, suggesting they function as potential suppressors of tumor formation. The expression of the remaining 96 dysregulated miRNAs showed increased expression as the tumorigenicity increased indicating they function as oncomiRs. The frequency distribution of the log transformed expression is displayed in Figure 4B. Of the 186 dysregulated miRNAs, only 65 miRNAs exhibited higher expression differences (≥ 8.0) as the phenotype of the cell changed.

**Figure 5:**
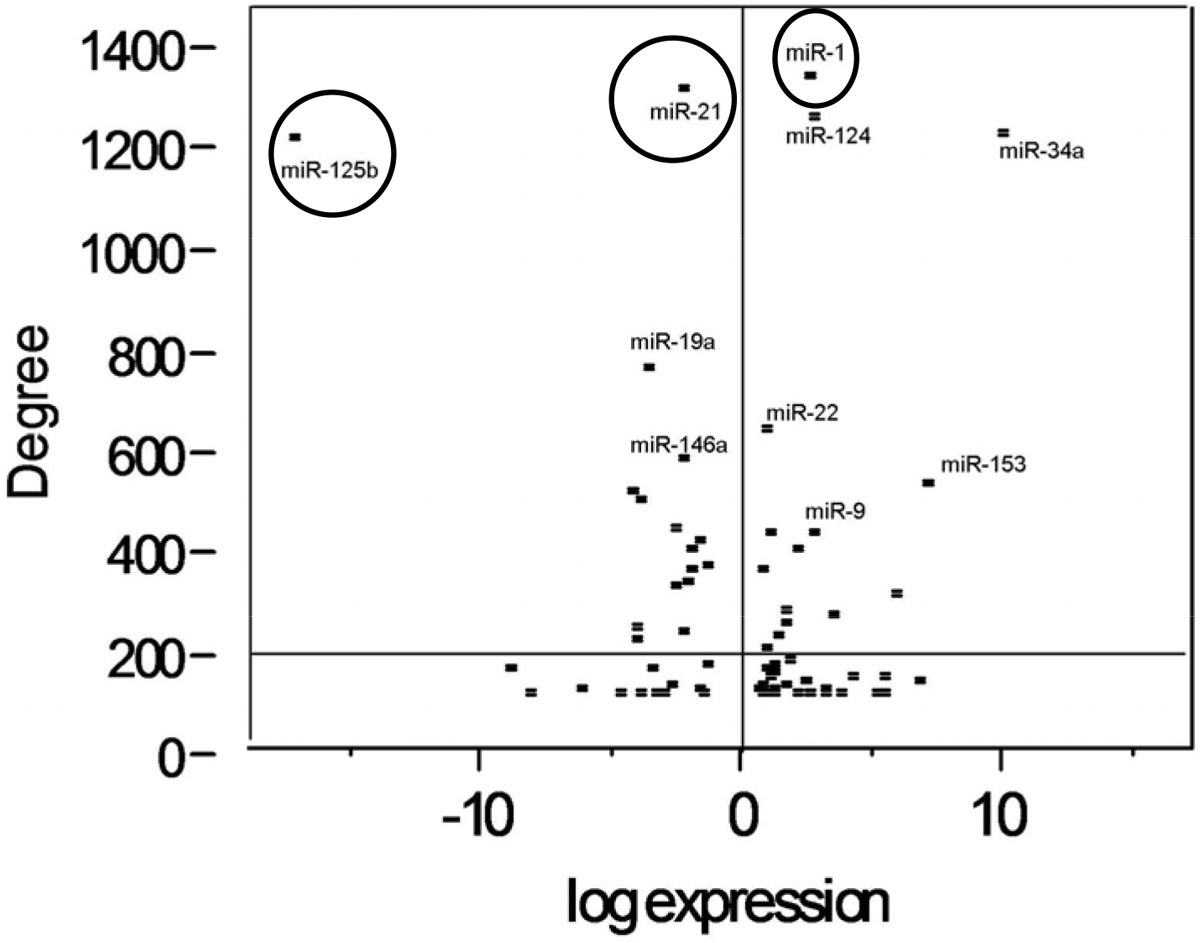
**Plot of changes in miRNA expression versus node degree**. Each dysregulated miRNA was plotted with the value of the X-axis being the log transformed fold change observed when comparing the M12 cells to the P69 cells. The y-axis represents the sum node degree of all experimentally verified targets of that miRNA. A horizontal line was drawn at 200, which is the mean node degree of all dysregulated miRNA targets. miRNAs with proven roles in cancer progression are identified with a circle whereas miRNAs identified with a square have an unproven role in tumor progression.

The cost to confirm every potentially dysregulated miRNA identified in a microarray experiment is prohibitively expensive. Few labs have the financial or physical resources needed to carry out such validation. Therefore, labs generally choose a subset of miRNAs to validate. Many factors affect the choice of a gene set including relative expression differences, biological function, availability of reagents, and investigator preference. Traditionally researchers have focused their efforts on extremely dysregulated miRNAs. This approach may overestimate the importance of differential expression. It is reasonable to suspect that smaller changes in some miRNAs may exert a greater influence in tissue behavior; i.e. if they modulate the expression of more important proteins. Many of the miRNAs that are altered during tumorigenesis changed between 2-8 fold. If one only focuses on the extremely dysregulated miRNAs, the contributions of many miRNAs will be overlooked. As noted in Figure 4, less than 20% of miRNAs are dysregulated greater than 8-fold. Most of the miRNAs change between 2-8 fold; therefore, if one only focuses on the extreme expression variances, the contributions of a large number of miRNAs will be overlooked. Based on the observation discussed earlier using known dysregulated miRNAs, our approach considers differential expression and functional relevance as ranked by the total node degree for all known protein targets. It is important to consider that not all miRNA targets are known but as the field evolves our method will remain applicable. miRNAs that exhibit greater than 2-fold expression differences between the non-tumorigenic and the tumorigenic, metastatic cell line were plotted against the total node degree of all proven interactions (Figure 5). The average connectivity of differentially expressed miRNAs is ~200 and indicated by a horizontal line dividing the graph into two regions. The points located near the top of the plot represent miRNAs that are proven to regulate important protein nodes that may play key roles in oncogenesis. A vertical line is drawn at 0, points located to the right function as oncomiRs in our cells and points to the left serve as potential suppressors of tumor formation. Several of these miRNAs have been previously described in cancer as discussed below but their role in prostate cancer development remains unresolved. The top 25 dysregulated miRNAs as ranked by the total node degree of proven protein targets is included in Table 3. Since, the purpose of this paper is not to elucidate miRNAs that drive prostate cancer development but rather outline a method for ranking differentially expressed miRNAs further biological and biochemical analyses are needed to define their contribution to the development of prostate cancer.

**Table 3:** 

miRNA ID	Fold Difference	Listed in miR2Disease	Node Degree of Proven Targets
hsa-miR-1	8.53	No	1330

hsa-miR-21	0.26	Yes	1302

hsa-miR-124	9.48	No	1242

hsa-miR-34a	1850.82	Yes	1208

hsa-miR-125b	0.00	Yes	1194

hsa-miR-19a	0.09	No	701

hsa-miR-22	2.67	Yes	565

hsa-miR-146a	0.26	Yes	504

hsa-miR-153	240.02	No	451

hsa-miR-20b	0.06	No	432

hsa-miR-15b	0.08	No	411

hsa-miR-100	0.21	Yes	354

hsa-miR-29c	2.75	No	339

hsa-miR-9	9.56	No	338

hsa-miR-181b	0.41	Yes	328

hsa-miR-99a	6.08	Yes	311

hsa-miR-31	0.32	Yes	305

hsa-let-7a	0.48	Yes	267

hsa-miR-27b	0.46	Yes	263

hsa-miR-7	2.38	No	263

hsa-miR-296-5p	0.31	No	260

hsa-miR-27a	0.30	Yes	234

hsa-miR-185	0.21	No	230

hsa-miR-133a	92.91	No	211

hsa-miR-181c	4.72	No	172

### Potential miRNAs regulating prostate tumorigenesis

Our method identified five miRNAs that clearly stood out from the others with total node degrees exceeding 1000. A review of the literature supports the fact that each of these may play a role in the tumorigenic propensity of the M12 cells (Figure 5). Three miRNAs (hsa-miR-125b, hsa-miR-1, and hsa-miR-21) with known roles in prostate cancer were identified (Figure 5 circled). Of these, one miRNA (hsa-miR-125b) is highly dysregulated during prostate cancer progression and likely would have been selected for further analyses based upon extreme differential expression. The other two (hsa-miR-1 and hsa-miR-21) are not as greatly dysregulated and their contribution to oncogenesis may have been overlooked. Potential roles in cancer development for these selected miRNAs are discussed below. Two miRNAs (hsa-miR-124 and hsa-miR-34a) may play a role in prostate cancer progression but their mechanism is not as clearly defined and additional investigation is needed. They are identified in Figure 5 with a square.

Human miR-125b was greatly decreased in the highly tumorigenic, metastatic M12 cell line. There are many proven targets of miR-125b including the epidermal growth factor receptors (EGFR) ErbB2 and ErbB3 [21]. Increased levels of ErbB2/ ErbB3 can lead to uncontrolled cellular proliferation and inhibition of apoptosis through activation of the AKT pathway. Patients with metastatic, hormone-refractory prostate cancer all showed an increase in EGFR expression and overexpression of the EGFR receptor has been shown to be associated with poor outcomes [22,23]. A recent study that compared matched prostate tumorigenic epithelium to benign epithelium revealed significant down regulation of miR-125b suggesting that the decreased expression of miR-125b can be used as a potential biomarker to discern malignant from benign epithelium.[24]. Increased expression of miR-125b in highly tumorigenic and metastatic prostate cancer cells may decrease tumorigenicity by inhibiting the EGFR family of growth factor receptors also making miR-125b an attractive therapeutic target. In this cell progression model, miR-125b clearly functions as a tumor suppressor.

miR-21 possesses diverse roles in cell proliferation, invasion and motility [25]. Many studies have shown that miR-21 functions as an oncomiR. It often increases during the process of tumorigenesis targeting a large number of genes that inhibit tumorigenic transformation [26]. However, a recent study by Folini *et al*. shows that many prostate cancer patients suffer a down regulation of miR-21 [27]. This suggests a need to consider any dysregulated miRNA in the context of the disease as its role could change depending upon the tissue source. miR-21 is not the only miRNA to display contrasting behaviors during cancer development. Eleven miRNAs have shown conflicting results during prostate tumor progression and can function as either a tumor suppressor or an oncomiR [13]. Despite the conflict, miR-21 remains an interesting target that needs further investigation.

Predominantly thought to induce cardiac/skeletal muscle differentiation and development, miR-1 also increases during the development of prostate cancer [28]. Dozens of targets have been proven to be regulated by miR-1, the most well described of which is HDAC4, a histone deacetylase. Androgen insensitivity is commonly observed in most disseminated prostate carcinomas. Localization of HDAC4 in the nucleus of androgen insensitive cancer cell lines was observed and hypothesized to contribute to the development of the hormone refractive phenotype [29]. HDAC4 represses transcription of the androgen receptor [30]. It remains probable that miR-1 may be an important oncomiR that drives prostate cancer progression justifying further investigation.

Although a role for the remaining two miRNAs (hsa-miR-34a and hsa-miR-124) is less obvious, a case can be made for their possible role in prostate tumorigenesis. Studies have demonstrated that the decreased expression of miR-34a may enhance cancer progression [31]. Wild type P53 has been shown to transactivate miR-34a. It is known that PC3 and DU145 cells are null or express a mutant P53 respectively when compared to the LnCap cells that express wild type P53. Thus miR-34a expression is dependent upon a secondary regulator. Although the status of P53 needs to be investigated in the cell lines, it is possible that P53 expression could account for differences in miR-34a levels. miR-124 is enriched in brain tissue and its over expression in neural stem cells induces differentiation [32]. Other studies have shown that miR-124 functions as a tumor suppressor and is lost during tumorigenesis [33,34]. As mentioned previously, several miRNAs have shown contrasting roles and therefore conflicting roles must be considered between different types and stages of carcinoma progression. In our cell model the expression of miR-124 increases during tumorigenesis. Although clear roles for these miRNAs in prostate tumor progression could not be identified, our study would suggest their investigation is warranted.

## Conclusion

The method described here extends a researcher's ability to not only consider the extent of differential expression but includes consideration of functional importance when choosing which miRNAs to further characterize. This work shows that miRNAs preferentially target messages that are highly connected and when aberrantly expressed results in a loss of cell cycle control leading to increased proliferation, invasion and metastasis. Expression profiling of a prostate cancer cell progression model revealed a large number of dysregulated miRNAs, several of which have known roles in the development of cancer (miR-125b, miR-21 and miR-1) but their involvement in prostate cancer progression was not clear. Two new miRNAs (miR-34a and miR-124) that likely affect prostate tumor progression were identified but a literature review does not indicate a clear role, and thus continued investigation is needed to prove their role in cancer formation.

## Materials and methods

### Protein-protein interaction network

Proven miRNA/gene interactions (2058) were assembled from Tarbase and miRecords [35,36]. Multiple entries were eliminated and resources combined into a single non-redundant data table. A list of d[14]ysregulated miRNAs contributing to prostate cancer were obtained from miR2disease and each associated with validated targets using our comprehensive record [37]. Transcriptome profiles from the Unigene database were obtained and used to determine miRNA targets expressed in the prostate. Known prostate miRNA targets were used to build a protein-protein interaction network [38]. An Agilent literature search (v2.76) tool installed in Cytoscape 2.8 was used to infer two protein-protein interaction networks [39,40]. A prostate cancer miRNA targeted network was inferred from a candidate list of 608 known prostate cancer miRNA target proteins. Each protein was used as a search term in the Agilent literature search tool and the search controlled to limit interactions to Homo sapiens with a maximum of 10 hits per search string/ search engine. As a control, the second network was built in the same manner using 608 randomly chosen proteins that are expressed in the prostate but chosen without regard to known miRNA status [38]. Following network inference, visualization was accomplished using Cytoscape and topological network descriptors were estimated using CentiScaPe [41].

### Pathway analysis

The two lists of proteins were uploaded into the ToppCluster gene enrichment analyzer, each as a cluster [15]. The first set was composed of the validated protein targets regulated by miRNAs (608) and the second, the list of randomly sampled and expressed prostate proteins. Multiple comparisons were corrected using a Bonferroni correction and statistical significance was set at p-value < 0.05. Data is presented as the log transformed p-value at base 10.

### Cell culture

Cells were cultured at 37° C in RPMI1640 with L-glutamine obtained from Gibco supplemented with 5% fetal bovine serum, 5 μg/ml insulin, 5 μg/ml transferrin, and 5 μg/ml of selenium (ITS from Collaborative Research Bedford, MA). Inhibition of bacterial contamination was accomplished with the addition of Gentamycin (0.05 mg/ml). All tissue culture cells were grown in 75 cm^2 ^flasks and split when 60-70% confluent. Cells were pelleted after trypsin (0.25% in EDTA) digestion and inactivation with serum-containing media by centrifugation at 5000 RPM for five minutes. After washing, cell pellets were flash frozen in liquid nitrogen and stored for at least 24 hours.

### Cell pellet RNA extraction

Total RNA was extracted from cell pellets described above using the miRVana™ miRNA isolation kit from Ambion per manufacturer's instructions. Briefly after cell lysis and organic extraction, total RNA was bound to a glass fiber filter, washed and eluted with a proprietary elution buffer. After isolation, RNA concentration was estimated using a Biorad^® ^Smart Spec™ 3000 spectrophotometer, diluted to a concentration of 100 ng/μl and stored at -80°C for at least 24 hours.

### MicroRNA profiling

Real time PCR profiling was performed using the miRCURY LNA ™ Universal RT microRNA PCR system (Exiqon, Denmark). Human Panel I was used to identify dysregulated miRNAs in the P69 cell line versus its metastatic derivative M12. Duplicate samples were compared using 25 ng and 50 ng of RNA as input. RNA input was converted to cDNA using supplied reagents and enzymes (4 μl of 5x reaction buffer, 9 μl of nuclease free water, 2 μl enzyme mix, 1 μl synthetic RNA spike in, and 5 μl of RNA diluted to 5 ng/ul). The reaction was incubated for 60 minutes at 42° C and the enzyme was heat inactivated for 5 minutes at 95°C. Real time PCR plates were run on an ABI7900 HT (95°C for 10 mins, 40 cycles at 95°C for 10 secs, for 60°C-1 min, ramp rate 1.6°C/s). Threshold and baseline were set manually according to recommendations in the supplied protocol. After correcting for interplate variability, cycle threshold (Ct) values were normalized to the global mean expression of all miRNAs. Initial data analysis was performed using Exiqon GenEx software and all values are reported as fold changes relative to P69. miRNAs exhibiting greater than 2-fold expression differences in both sets of arrays were considered to be significant and selected for further analysis. The data discussed in this publication has been submitted in NCBI's Gene Expression Omnibus and accessible through GEO as accession number GSE49520. [42].

### Statistical analysis

Differences in network distributions were evaluated using an Analysis of Variance test (ANOVA) with significance set at probability ≤ 0.05. All statistical analyses were performed using JMP 8.0 (Statistical Analysis Software Cary, NC). Figures 1 and 5 were created using the R Project for Statistical Computing (http://www.r-project.org). The remaining data analyses and figures were compiled and constructed using Microsoft Excel.

## List of abbreviations used

miRNA    microRNA

mRNA    messenger RNA

PPI    protein protein interaction network

EGFR    epidermal growth factor receptor

## Competing interests

The authors declare that they have no competing interests.

## Authors' contributions

WTB and DW conceived and designed the method utilized in this work. WTB and SS carried out the miRNA expression profiling. DW compiled the list of known miRNAs in prostate cancer and built the protein-protein interaction network. CJ assisted with analysis of the miRNA expression data and assisted with the drafting of the manuscript. All of the work took place in the lab of ZEZ under her direct supervision and guidance. All of the authors have read and approved the final manuscript.

## Declarations

The publication costs for this paper were covered by Award Number R21CA152349 to ZEZ.

This article has been published as part of *BMC Systems Biology *Volume 7 Supplement 5, 2013: Selected articles from the International Conference on Intelligent Biology and Medicine (ICIBM 2013): Systems Biology. The full contents of the supplement are available online at http://www.biomedcentral.com/bmcsystbiol/supplements/7/S5.
